# Epigenetic-Based Biomarkers in the Malignant Transformation of BEAS-2B Cells Induced by Coal Tar Pitch Extract

**DOI:** 10.3390/medicina57010024

**Published:** 2020-12-29

**Authors:** Shuyin Duan, Huijie Yuan, Songcheng Yu, Xiaoling Wei, Xiaoshan Zhou, Wei Wang, Feifei Feng, Lingbo Qu, Yongjun Wu

**Affiliations:** 1 Department of Occupational and Environmental Health, College of Public Health, Zhengzhou University, Zhengzhou 450001, China; shuyinduan@126.com (S.D.); Xiaoshan.Zhou@ki.se (X.Z.); ww375@126.com (W.W.); 2 Department of Toxicology, College of Public Health, Zhengzhou University, Zhengzhou 450001, China; YHJLOVE2013@163.com (H.Y.); zzuhappy2013@126.com (X.W.); heartnessff@126.com (F.F.); 3 Department of Sanitary Chemistry, College of Public Health, Zhengzhou University, Zhengzhou 450001, China; scyu@zzu.edu.cn; 4 Henan Joint International Research Laboratory of Green Construction of Functional Molecules and Their Bioanalytical Applications, Zhengzhou University, Zhengzhou 450001, China; zhengdacpu@163.com

**Keywords:** coal tar pitch extract, BEAS-2B cells, epigenetics, DNA methylation, occupational lung cancer, biomarkers

## Abstract

*Background and Objectives:* The carcinogenicity of coal tar pitch (CTP) to occupational workers has been confirmed by the International Agency for Research on Cancer, especially for lung cancer. Herein, we explored the dynamic changes of epigenetic modifications in the malignant transformation process of CTP-induced BEAS-2B cells and also provided clues for screening early biomarkers of CTP-associated occupational lung cancer. *Material and Methods:* BEAS-2B cells treated with 3.0 μg/mL CTP extract (CTPE) were cultured to the 30th passage to set up a malignant transformation model, which was confirmed by platelet clone formation assay and xenograft assay. DNA methylation levels were determined by ultraviolet-high performance liquid chromatography. mRNA levels in cells and protein levels in supernatants were respectively detected by Real-Time PCR and enzyme-linked immunosorbent assay. *Results:* The number of clones and the ability of tumor formation in nude mice of CTPE-exposed BEAS-2B cells at 30th passage were significantly increased compared to vehicle control. Moreover, genomic DNA methylation level was down-regulated. The mRNA levels of DNMT1, DNMT3a and HDAC1 as well as the expression of DNMT1 protein were up-regulated since the 10th passage. From the 20th passage, the transcriptional levels of DNMT3b, let-7a and the expression of DNMT3a, DNMT3b, and HDAC1 proteins were detected to be higher than vehicle control, while the level of miR-21 increased only at the 30th passage. *Conclusion:* Data in this study indicated that the changes of epigenetic molecules including DNMT1, DNMT3a, DNMT3b, HDAC1, and let-7a occurred at the early stages of BEAS-2B cell malignant transformation after CTPE exposure, which provided critical information for screening early biomarkers of CTP-associated occupational lung cancer.

## 1. Introduction

Asphalt laborers, such as coke oven workers and road-menders, are highly exposed to coal tar pitch (CTP), a by-product obtained from coal-fired and coking process. CTP is mainly composed of polycyclic aromatic hydrocarbons and has been confirmed as a carcinogen by the International Agency for Research on Cancer (IARC), especially for lung cancer [1,2]. Lung cancer induced by CTP has been classified as occupational lung cancer, and has become an important health issue in occupational population. An epidemiological survey has shown that the risk of lung cancer in coke oven workers is 16 times higher than those in the normal control, and this is closely related to the working hours of coke oven workers [3]. As reported by the National Cancer Statistics of United States in 2018, the five-year survival rate for lung and bronchus cancer decreased from 55.1% of stage I to 4.2% of stage IV [4]. However, only 25.3% of lung and bronchus cancer patients were diagnosed at stage I or stage II while 66.9% of cases were diagnosed at stage III or stage IV due to the lack of early diagnostic biomarkers [4]. Therefore, the key way to reduce the risk associated with occupational lung cancer is to find effective and early biomarkers, which will contribute to early screening, early diagnosis and early treatment of lung cancer.

Occurrence of lung cancer is an integrated biological process involving genetics, epigenetics, and environmental factors. Genetic effects, such as DNA damage, chromosomal instability, and telomere damage that are induced by CTPE (CTP extract), have been confirmed in our previous researches [5,6]. However, epigenetic changes in this process have not been studied in depth. Epigenetics is not involved in the changes of DNA sequence, but it regulates the activation and silencing of genes through changing the structure of chromatin and affecting the binding of transcription factors to DNA [7]. Moreover, the abnormal changes in epigenetics appear earlier than the evident malignant phenotypes and can be reversed easily [8]. Therefore, epigenetics could provide a new insight in the screening of biomarkers, and this contribute to the identification of early, reversible and effective biomarkers for CTP-associated occupational lung cancer.

DNA methylation, histone modifications and miRNAs in epigenetics have gradually become the hotspot of research in recent years. According to reports, the interaction between DNA methylation and histone modification could induce gene silencing, because methylated DNA can be converted into dense chromatin under the action of histone deacetylases (HDACs) and DNA methyltransferases (DNMTs), which results in transcriptional repression [9,10]. In addition, DNA methylation can also interact with miRNAs to regulate the development of cancer [11,12]. miR-21 and let-7a are hypermethylated in normal human tissues, but hypomethylation of these in cancer tissues promotes the expression of miR-21 and let-7a, which is related to the malignant transformation progress of tumors [13,14,15]. However, most of the reported studies were based on clinically advanced cancer samples, and the dynamical changes of those epigenetic molecules in the early stages of lung cancer have never been systematically tracked. Thus, it is still obscure whether such molecules can be used as effective early biomarkers for CTP-associated occupational lung cancer.

In this study, we established a malignant transformation model in vitro after BEAS-2B cells were stimulated with CTPE. The purpose of our present study was to track the dynamic changes of epigenetic molecules including DNA methyltransferase1 (DNMT1), DNA methyltransferase3a (DNMT3a), DNA methyltransferase3b (DNMT3b), histone deacetylase enzyme1 (HDAC1), as well as miR-21 and let-7a during the malignant transformation process of CTPE-induced BEAS-2B cells. This study would provide useful clues for screening early biomarkers of CTP-associated occupational lung cancer.

## 2. Materials and Methods

### 2.1. Preparation of Coal Tar Pitch Extract

Coal tar pitch at moderate temperature was collected from an aluminum company in Henan Province, China. They were grinded into powder, sieved by 200 meshes of 0.074 mm in diameter and heated at 400 °C to collect fume on 0.8 μm nitrocellulose membranes with a dust sampler. Then, the membranes were cut into pieces, placed in a stoppered flask and dissolved with ethyl acetate solution. Finally, the solution was filtered through a sand core funnel and dried at 45 °C. CTPE was dissolved in dimethyl sulfoxide (DMSO) to a concentration of 2.0 mg/mL and stored at 4 °C for future use. The components of CTPE were analyzed using gas chromatography and mass spectrometry (GC/MS), and the related components could refer to our previous research [1]. 

### 2.2. Cell Culture and CTPE Treatment

The BEAS-2B cell line was purchased from BeiNa Culture Collection (Beijing, China) and cultured in RPMI 1640 medium containing 10% (*v*:*v*) fetal bovine serum (FBS) at 37 °C in a 5% CO_2_ incubator. When the cells reached 70–80% confluency, BEAS-2B cells were treated with 3.0 μg/mL CTPE (30% of the IC50 [16]) for 24 h. Then, they were washed with 1× PBS for 3 times and passaged using trypsin-EDTA. The cells were treated 3 times as described above and then cultured with normal medium. The first passage of cells after being treated 3 times was denoted as passage 1 and the cells were cultured to passage 30. DMSO served as a vehicle control and 5.0 μg/mL benzo(a)pyrene (B(a)P) [16] was used as a positive control.

### 2.3. Plate Clone Formation Assay

100 cells/well at passage10, 20, or 30 in the DMSO, CTPE or B(a)P group were seeded into a 6-well plate and incubated for 2 weeks at 37 °C in 5% CO_2_. The medium was changed every 5 days and the clone of more than 50 cells was counted after being fixed with 95% methanol for 15 min and stained with Giemsa stain for 10 min.

### 2.4. Xenograft Assay

Three to four week old male Balb/c nude mice were purchased from Hunan Slack King Laboratory Animal Co, Ltd. (Changsha, China). The experimental protocol for tumor formation in nude mice was approved by the Life Sciences Institutional Review Board of Zhengzhou University. Mice were treated humanely and with regard to relieve suffering. Firstly, 2 × 10^7^ of the cells at passage 30 in the various groups (DMSO, CTPE, and B(a)P groups) were suspended in 200 μL PBS and injected into the right flanks of each nude mouse. Tumor formation in the nude mice was closely observed and the mice were sacrificed by euthanasia on the 38th day, which referenced to our previous study [17].

### 2.5. Ultraviolet-High Performance Liquid Chromatography (UV-HPLC)

The total DNA from the cells at passage 10, 20, and 30 in DMSO, CTPE or B(a)P group was extracted using Universal Genomic DNA Extraction Kit (TaKaRa, Tokyo, Japan) respectively. DNA concentration was measured by micro nucleic acid analyzer. The total DNA was treated by a hydrolytic Kit (TaKaRa, Tokyo, Japan) following manufacturer’s instruction. Ultraviolet-high performance liquid chromatography (UV-HPLC) was used to determine the hydrolysate level of deoxycytidine(dc) and 5-methyl-2′-deoxycytidine (5-dmC). Under the optimizing experimental conditions, 0.8% (*v*/*v*) of tetrahydrofuran in 50 mmol/L of ammonium phosphate solution (pH 4.l) was used as mobile phase at a flow rate of 1 mL/min, 279 nm as the detection wavelength of dc and 5-dmc [18]. Based on the relationship between peak area and concentration, the linear regression equations of dC and 5-dmC were established respectively, and R values of their correlation coefficients were calculated. The global level of DNA methylation was expressed as the ratio of 5-dmc/(dc+5-dmc).

### 2.6. Real-Time PCR (RT-PCR)

RNAiso Plus kit (TaKaRa, Tokyo, Japan) and RNAiso kit for small RNAs (TaKaRa, Tokyo, Japan) were used to extract total RNA and small RNA from the cells, respectively. PrimeScript^®^ RT reagent Kit (TaKaRa, Tokyo, Japan) and PrimeScript miRNA cDNA Synthesis Kit (TaKaRa, Tokyo, Japan) were applied to convert RNA into cDNA. The mRNA levels of DNMT1, DNMT3a, DNMT3b, HDAC1, let-7a, and miR-21 were determined using the MX3000P QPCR System (Stratagene, San Diego, CA, USA) with SYBR^®^ Priemix Ex Taq^TM^ II kit (TaKaRa, Tokyo, Japan). GAPDH was used as a standard control for DNMT1, DNMT3a, DNMT3b and HDAC1. miR-21 and let-7a were normalized by snRNA U6. All operations were performed according to the instructions of the manufacturers. The primer sequences (Sangon Biotech, Shanghai, China) for GAPDH, snRNA U6 and the target genes were as follows, GAPDH: Forward primer, 5′-TACTAGCGGTTTTACGGGCG-3′ and Reverse primer, 5′-GAACAGGAGGAGCAGAGAGCGA-3′. snRNAU6: Forward primer, 5′-CGATTGGAACGATACAGAGAAGAT-3′. let-7a: Forward primer, 5′-CGGTGAGGTAGTAGGTTGTATAGTT-3′. miR-21: Forward primer, 5′-GGGACTAGCTTATCAGACTGAAA-3′. DNMT1: Forward primer, 5′-CGACTACATCAAAGGCAGCA-3′ and Reverse primer, 5′-TGGACTTGTGGGTGTTCTCA-3′. DNMT3a: Forward primer, 5′-CCCGAAGGTTTACCCACCTG-3′ and Reverse primer, 5′-GCGATGTAGCGGTCCACTTG-3′. DNMT3b: Forward primer, 5′-GAATGCGCTGGGTACAGTGG-3′ and Reverse primer, 5′-GCCAGATTAAAGTGCTGGCTGA-3′. HDAC1: Forward primer, 5′-AGTGCGGTGGTCTTACAGTG-3′ and Reverse primer, 5′-CCTCCCAGCATCAGCATA G-3′.

### 2.7. Elisa

The culture supernatants of the cells at passage 10, 20, and 30 in DMSO, CTPE, and B(a)P groups were collected and centrifuged at 1500g for 20 min at 4 °C. The protein levels of DNMT1, DNMT3a, DNMT3b, and HDAC1 in the supernatant were determined using Elisa kits (Cusabio Biotechnology, Wuhan, China).

### 2.8. Statistical Analysis

Statistical analysis was performed using SPSS 21.0 (IBM, Armonk, NY, USA) and results were expressed as mean ± standard deviation. One-way ANOVA was employed to determine the differences among various groups, and the differences between every two groups were then compared using LSD. A *p*-value < 0.05 was considered statistically significant.

## 3. Results

### 3.1. BEAS-2B Cell Canceration Following CTPE Exposure Was Confirmed by Clone Formation in Plate and Tumorigenesis in Nude Mice

**Figure 1:**
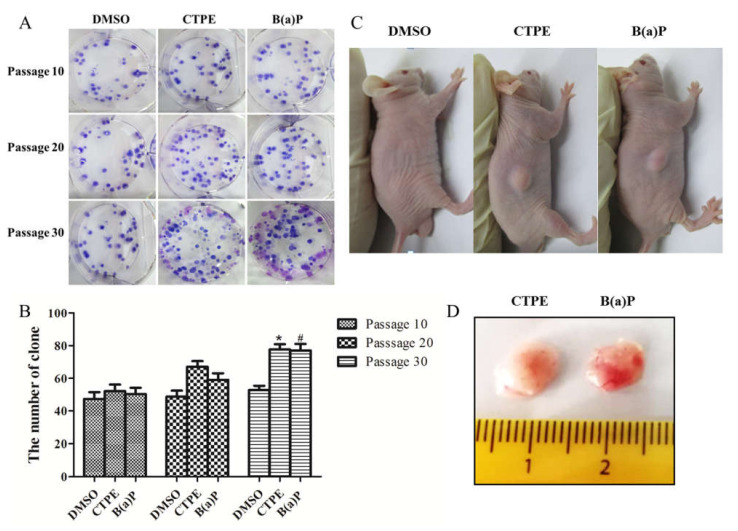
Clone formation in plate and tumor formation in nude mice using cells at passage 30. (**A**) Representative clones in different passages and groups. (**B**) The number of clones in different passages and groups (*n* = 3, *: CTPE vs. DMSO, *p* < 0.05. #: B(a)P vs. DMSO, *p* < 0.05). (**C**,**D**) Tumor formations were observed in nude mice on the 38th day after injecting CTPE- or B(a)P-induced BEAS-2B cells at passage 30.

Plate clone formation assay was employed to evaluate the anchorage-independent growth capabilities of BEAS-2B cells. As shown in Figure 1A,B, the number of clones of BEAS-2B cells stimulated with CTPE or B(a)P was not statistically different from that of the DMSO group at passage 10 and passage 20 (*p* > 0.05). However, there was an obvious increase at passage 30 (*p* < 0.05). To further confirm the malignancy of CTPE-treated cells at passage 30, we initiated tumorigenesis experiment in the nude mice. Clearly, tumors were only observed in the nude mice injected with passage 30 of the CTPE- or B(a)P-treated BEAS-2B cells (Figure 1C,D). These results indicated that the malignant transformation model of cells that were induced by CTPE was successfully set up.

### 3.2. Genomic DNA Methylation Levels Were Down-Regulated in CTPE-Induced BEAS-2B Cells

**Figure 2:**
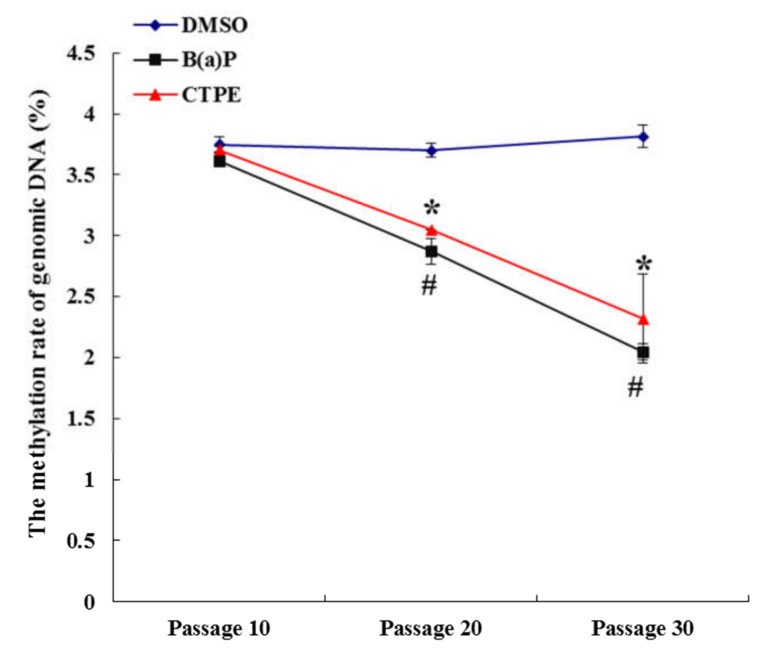
Methylation rates of genomic DNA in the cells from three groups. The methylation rates of genomic DNA in DMSO, CTPE, and B(a)P groups at different detection time points. The results were shown as mean ± SD (*n* = 3, *: CTPE vs. DMSO, *p* < 0.05. #: B(a)P vs. DMSO, *p* < 0.05).

As shown in Figure 2, there were no significant differences in methylation rates of genomic DNA at passage 10 (*p* > 0.05), but the methylation rates of cells decreased at passage 20 and passage 30 in the CTPE- or B(a)P-induced group compared with the DMSO group (*p* < 0.05). As the number of cell passages increased, the methylation rate in the CTPE or B(a)P group also decreased gradually and the differences were statistically significant (*p* < 0.05). However, there was no significant difference among the DMSO groups (*p* > 0.05).

### 3.3. mRNA Levels of DNMT1, DNMT3a, DNMT3b, and HDAC1 Were Up-Regulated in CTPE-Exposed BEAS-2B Cells

**Figure 3:**
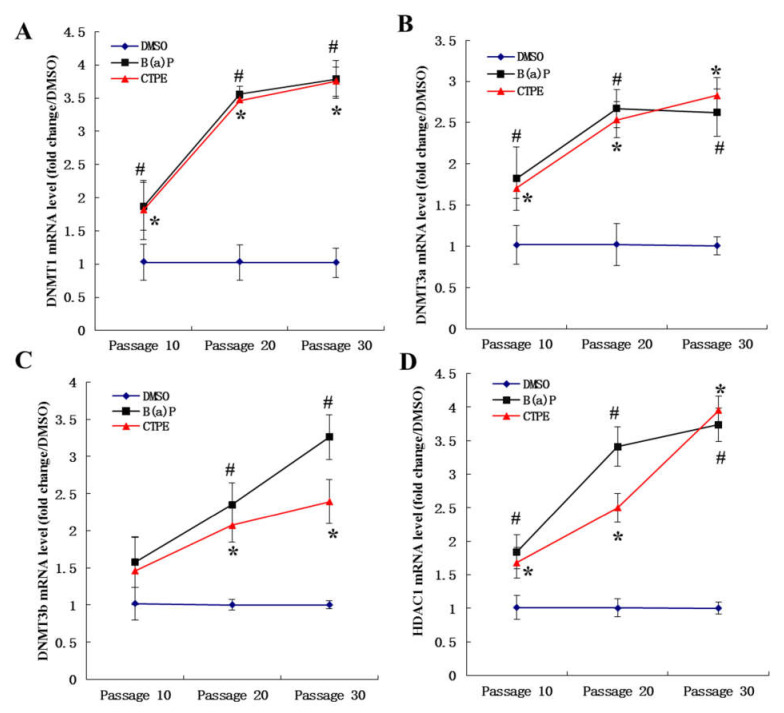
The mRNA levels of DNMT1, DNMT3a, DNMT3b, and HDAC1 of cells in the three groups. The mRNA levels of (**A**) DNMT1, (**B**) DNMT3a, (**C**) DNMT3b or (**D**) HDAC1 in DMSO, CTPE, and B(a)P groups at passage 10, 20 and 30. The results were presented as mean ± SD (*n* = 3, *: CTPE vs. DMSO, *p* < 0.05. #: B(a)P vs. DMSO, *p* < 0.05).

Levels of key enzymes that regulate DNA methylation were evaluated by RT-PCR. The mRNA levels of DNMT1 (Figure 3A), DNMT3a (Figure 3B), and HDAC1 (Figure 3D) in the cells of passage 10, 20, and 30 that were exposed to CTPE or B(a)P were significantly higher than those in the DMSO group (*p* < 0.05). However, there was no significant difference in the level of DNMT3b mRNA among three groups at the 10th passage (*p* > 0.05). The mRNA levels of DNMT3b were significantly increased in the CTPE and B(a)P groups at the 20th and 30th passage (*p* < 0.05). With the increase of passage, the mRNA levels of DNMT1, DNMT3a, DNMT3b, and HDAC1 also increased in the CTPE- or B(a)P-stimulated BEAS-2B cells (*p* < 0.05). However, there was no significant difference in the DMSO group among different passages (*p* > 0.05).

### 3.4. The Protein Levels of DNMT1, DNMT3a, DNMT3b, and HDAC1 Increased in the Culture Supernatants of CTPE-Stimulated BEAS-2B Cells

**Figure 4:**
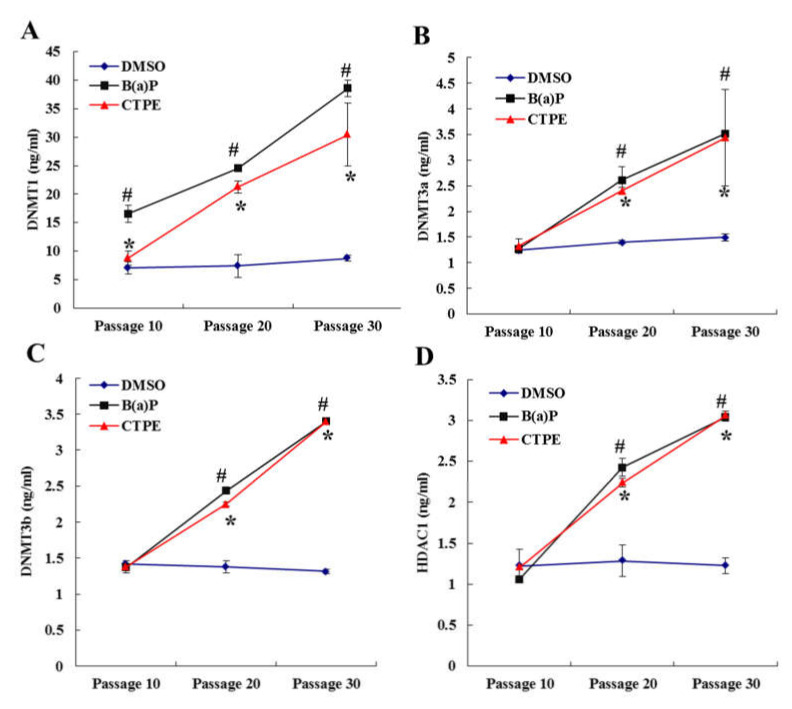
The protein levels of DNMT1, DNMT3a, DNMT3b, and HDAC1 in the culture supernatants. The protein levels of (**A**) DNMT1, (**B**) DNMT3a, (**C**) DNMT3b or (**D**) HDAC1 in cell supernatants of DMSO, CTPE, and B(a)P groups at passage 10, 20 and 30. The results were presented as mean ± SD (*n* = 3, *: CTPE vs. DMSO, *p* < 0.05. #: B(a)P vs. DMSO, *p* < 0.05).

As shown in Figure 4A, the levels of DNMT1 protein in the culture supernatants of CTPE and B(a)P groups were higher than those in the DMSO group at passage 10, 20, and 30 (*p* < 0.05). There were no significant differences in the levels of DNMT3a (Figure 4B), DNMT3b (Figure 4C) and HDCA1 (Figure 4D) among the three groups at passage 10 (*p* > 0.05), but a significant increase was seen in the CTPE and B(a)P groups at passage 20 and 30 (*p* < 0.05). The protein levels of DNMT1, DNMT3a, DNMT3b, and HDCA1 in the cell culture supernatants were not significantly different in the DMSO group as the number of cell passages increased (*p* > 0.05), but they were significantly increased in the CTPE and B(a)P groups (*p* < 0.05).

### 3.5. let-7a and miR-21 Were Detected to be Highly Expressed in CTPE-Treated BEAS-2B Cells

**Figure 5:**
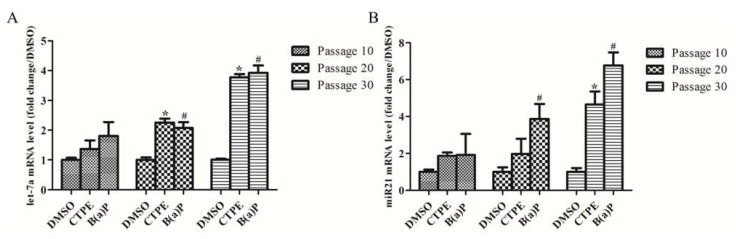
let-7a and miR-21 levels in cells at passage 10, 20, and 30 among three groups. The levels of (**A**) let-7a and (**B**) miR-21 in DMSO, CTPE, and B(a)P groups at passage 10, 20, and 30. The results were presented as mean ± SD (*n* = 3, *: CTPE vs. DMSO, *p* < 0.05. #: B(a)P vs. DMSO, *p* <0.05).

As shown in Figure 5, the levels of let-7a and miR-21 in the CTPE- or B(a)P-induced BEAS-2B cells increased gradually as the number of cell passages increased (*p* < 0.05). However, there was no significant difference at passage 10 among the three groups (*p* > 0.05). Moreover, the levels of let-7a (Figure 5A) in CTPE- or B(a)P-induced cells were significantly higher than that in the DMSO group since passage 20, while the miR-21 (Figure 5B) levels in the CTPE and DMSO groups were significantly different only at passage 30 (*p* < 0.05).

## 4. Discussion

Traditionally, the diagnostic methods of lung cancer have certain limitations, which include high false positive rate in CT (computed tomography) examination, low sensitivity in sputum examination and severe trauma in bronchoscopic examinations. Many molecular events, such as epigenetic changes, occur earlier than the evident malignant phenotypes during the development of lung cancer and can be detected by molecular biology methods [7]. Molecular events are considered as the most promising means to detect precancerous lesions or early cancer. Epigenetic changes without involving the changes of DNA sequence appear early in tumor development and are reversible, which have gradually drawn the attention of the general public. Among them, DNA methylation, histone modification, and miRNA have become hot topics. In this study, CTPE was used to induce the malignant transformation of BEAS-2B cells, and the dynamic changes of epigenetic molecules in this process were tracked to provide clues for screening early biomarkers of CTP-associated occupational lung cancer.

As shown by the clone formation assay and xenograft assay, the 20th passage cells, after being exposed to CTPE, were in the process of malignant transformation and the 30th passage cells had completely malignant transformation. Here, we further confirmed that the genomic DNA methylation rate of CTPE-exposed cells decreased along with the increasing malignancy. The results implied that the down-regulation of genomic DNA methylation played an important role in the malignant transformation of the cells, which was consistent with the report by Ahmad [19]. It was known that DNA methylation was mediated by DNMTs and HDACs [20]. The biologically active DNMTs mainly include DNMT1, DNMT3a, and DNMT3b. DNMT1 is able to maintain the DNA methylation status, while DNMT3a and DNMT3b participate in methylation process [21]. Some reports demonstrated that the abnormal expression of DNMTs preceded the aberration of methylation level, which were the early molecular events of tumors [22]. Notably, the paradoxical role of DNMT3a that can act both as an oncogene and as a tumor suppressor gene in lung cancer has been proposed in a recent review [23]. Our current study demonstrated that the mRNA levels in cells and the protein levels in the supernatants of DNMT1, DNMT3a, DNMT3b, and HDAC1 increased with the increase of cell malignancy, which has provided a critical evidence in the search for tumor biomarkers from body fluid [24]. Similarly, it was reported that increased expression of DNMT3a led to the proliferation and metastasis of lung cancer cells [23]. Among them, the levels of DNMT1 mRNA and protein were higher in the CTPE or B(a)P group than in the DMSO group since passage 10, suggesting that DNMT1 changed at the early stage of malignant transformation of the BEAS-2B cells. The mRNA levels of DNMT3a and HDAC1 in CTPE and B(a)P groups were statistically different from that of the DMSO group since passage 10 and the similar results at protein levels were also found from passage 20. However, the mRNA and protein levels of DNMT3b were higher in the CTPE and B(a)P groups than those in the DMSO group since passage 20. In summary, we found that DNMT1 was more sensitive than DNMT3a and HDAC1, while DNMT3a and HDAC1 were more sensitive than DNMT3b in predicting the malignant transformation of CTPE-induced BEAS-2B cell.

Clinical studies using RT-PCR and chip technology have confirmed the differential expression of let-7a and miR-21 among advanced lung cancer and normal control [13,14,15,25,26]. Our current studies, however, focused on the dynamic changes of these in the malignant process of CTPE-induced BEAS-2B cell. The results showed that the gene expression levels of let-7a and miR-21 increased along with the increase of cell malignancy. The level of let-7a in the CTPE group was higher than in the DMSO group since passage 20, whereas the differential expression of miR-21 could not be detected until the 30th passage. These results demonstrated that let-7a may serve as an early biomarker of BEAS-2B malignant transformation upon CTPE exposure, and that, miR-21 was not enough to prompt early malignant transformation.

In summary, we confirmed that malignant transformation of BEAS-2B cells induced by CTPE was associated with the hypomethylation of genomic DNA and the overexpression of DNMTs, HDAC1, let-7a, and miR-21. The epigenetic molecules of DNMT1, DNMT3a, DNMT3b, HDAC1, and let-7a might be considered as early biomarkers for the malignant transformation of CTPE-induced BEAS-2B cells. In future, the changes of those epigenetic molecules will be further explored using CTPE-induced lung cancer mice model. In totality, our study has provided critical clues in the screening of early biomarkers for CTP-associated occupational lung cancer.

## 5. Conclusions

In this study, we established a malignant transformation model in vitro and tracked the dynamic changes of epigenetic molecules. As a result, we found that the gene levels of DNMT1, DNMT3a, and HDAC1 in the cells as well as DNMT1 protein level in the culture supernatant were up-regulated since passage 10. The gene levels of DNMT3b, let-7a, and the proteins of DNMT3a, DNMT3b, and HDAC1 were detected to be elevated from passage 20, while the expression of miR-21 increased only at passage 30. Data in this study indicated that the changes of epigenetic molecules including DNMT1, DNMT3a, DNMT3b, HDAC1, and let-7a occurred at the early stages of BEAS-2B cell malignant transformation upon CTPE exposure, which has provided critical information on screening of early biomarkers for CTP-associated occupational lung cancer.

## Data Availability

The data of this study are available from the corresponding author upon reasonable request.
